# Postmastectomy Radiotherapy After Neoadjuvant Chemotherapy in cT_1-2_N_+_ Breast Cancer Patients: A Single Center Experience and Review of Current Literature

**DOI:** 10.3389/fonc.2022.881047

**Published:** 2022-05-17

**Authors:** Meng Luo, Huihui Chen, Hao Deng, Yao Jin, Gui Wang, Kun Zhang, Hong Ma, Yiding Chen, Suzhan Zhang, Jiaojiao Zhou

**Affiliations:** ^1^ Department of Breast Surgery and Oncology, the Second Affiliated Hospital, Zhejiang University School of Medicine, Hangzhou, China; ^2^ The Key Laboratory of Cancer Prevention and Intervention, China National Ministry of Education, Zhejiang University School of Medicine, Hangzhou, China; ^3^ Department of General Surgery, Longquan People’s Hospital, Longquan, China; ^4^ Cancer Center, Zhejiang University, Hangzhou, China

**Keywords:** postmastectomy radiotherapy, disease-free survival, overall survival, breast cancer, neoadjuvant chemotherapy

## Abstract

**Purpose:**

Postmastectomy radiotherapy (PMRT) after neoadjuvant chemotherapy (NAC) in breast cancer patients with initial clinical stage cT_1-2_N_+_, especially for those who achieved ypT_1-2_N_0_, is still controversial. This study was to evaluate the survival prognosis of cT_1-2_N_+_ patients after NAC with or without PMRT, and to discuss the selection of patients who may omit PMRT.

**Patients and Methods:**

From January 2005 to December 2017, 3055 female breast cancer patients underwent mastectomy in our medical center, among whom 215 patients of cT_1-2_N_+_ stage, receiving NAC with or without PMRT were finally analyzed. The median follow-up duration was 72.6 months. The primary endpoint was disease-free survival (DFS), and secondary endpoint was overall survival (OS). Comparison was conducted between PMRT and non-PMRT subgroups.

**Results:**

Of the 215 eligible patients, 35.8% (77/215) cT_1-2_N_+_ patients achieved ypT_0-2_N_0_ after NAC while 64.2% (138/215) of the patients remained nodal positive (ypT_0-2_N_+_). The 5-year DFS of ypT_0-2_N_0_ non-PMRT was 79.5% (95% confidence interval [CI] 63.4-95.6%). No statistically significant difference was observed between the ypT_0-2_N_0_ PMRT and non-PMRT subgroups for the 5-year DFS (78.5% vs 79.5%, *p* = 0.673) and OS (88.8% vs 90.8%, *p* = 0.721). The 5-years DFS didn’t obviously differ between the ypT_0-2_N_0_ non-PMRT subgroup and cT_1-2_N_0_ subgroup (79.5% vs 93.3%, *p* = 0.070). By using Cox regression model in multivariate analyses of prognosis in ypT_0-2_N_+_ PMRT subgroup, HER2 overexpression and triple-negative breast cancer were significantly poor predictors of DFS and OS, while ypN stage was significant independent predictors of OS.

**Conclusion:**

An effective response to NAC (ypT_0-2_N_0_) indicates a sufficiently favorable prognosis, and PMRT might be omitted for cT_1-2_N_+_ breast cancer patients with ypT_0-2_N_0_ after NAC.

## Introduction

Neoadjuvant chemotherapy (NAC) is increasingly used to treat patients with breast cancer, especially those with locally advanced disease (1). NAC can facilitate surgery by converting an inoperable tumor to an operable tumor or by converting a patient requiring mastectomy to one who can be treated with breast-conserving surgery (2). NAC usually alters tumor stage and has been found to downsize primary tumors in 70–80% of patients (3, 4). In addition, NAC was reported to downstage axillary lymph nodes status in 20–40% of patients (4, 5).

Postmastectomy radiotherapy (PMRT) has been shown to reduce the risk of locoregional recurrence and enhance overall survival in patients with breast cancer. PMRT is recommended for patients with tumors ≥ 5 cm size or those with at least four positive lymph nodes (6, 7). In contrast, the role of PMRT in T_1-2_ tumors with 1–3 positive lymph nodes remain unclear, with this treatment usually recommended for patients with T_1-2_N_1_ tumors and high-risk features. These guidelines for PMRT guidance, however, were formulated for patients in the absence of NAC, making the indications for PMRT unclear for patients who receive NAC. Moreover, the potential downstaging associated with NAC can alter the standard indications for PMRT.

Indications for PMRT following NAC remain unclear, particularly in patients initially diagnosed with stage cT_1-2_N_+_ breast tumors. Because increasing numbers of patients undergo breast reconstruction after surgery, determining indications for PMRT has become more important, as PMRT can adversely affect the incidence of complications and aesthetic outcomes of immediate breast reconstructions (8). Moreover, it has not yet been determined whether an effective response to NAC (e.g., achieve ypT_0-2_N_0_) can allow the omission of PMRT.

The present study evaluated the efficacy of PMRT after NAC and surgery in breast cancer patients with initial clinical stage cT_1-2_N_+_M_0_. Three questions were addressed: 1) whether patients presenting with cT_1-2_N_+_ disease who achieve ypT_0-2_N_0_ after NAC require PMRT; 2) the comparative prognosis in the absence of PMRT of patients with cT_1-2_N_+_ and cT_1-2_N_0_ who achieve ypT_0-2_N_0_ after NAC; and 3) the correlations between clinical variables and prognosis in patients with residual nodal disease after NAC. These findings may help determine the indications for PMRT after NAC.

## Methods

### Patient Population

Of the 3055 women diagnosed with breast cancer who underwent mastectomy at the Second Affiliated Hospital, Zhejiang University School of Medicine from January 2005 to December 2017, 456 (14.9%) received NAC before mastectomy. Patients who received fewer than two cycles of NAC (n=5), those with ypT_3-4_ stage after NAC (n=6), those with undetermined ypT stage (n=19) and ypN stage (n=1), and those with unknown radiotherapy (n=62) were excluded. This retrospective analysis therefore included 215 patients diagnosed with cT_1-2_N_+_ stage breast before NAC followed by mastectomy. The study protocol was approved by the Ethics Committee of our hospital (Approval No: 2020-363).

The medical records of all included patients were extracted from the computerized database of the Second Affiliated Hospital, Zhejiang University School of Medicine. Follow-up information on all patients was obtained by clinic visits and telephone contact. Clinical tumor size (cT) was determined by ultrasound, mammography or magnetic resonance imaging. Patients classified as cN_+_ were defined as those with clinically diagnosed metastatic lymph nodes, including palpable lymph nodes that were fixed or matted, metastatic lymph nodes diagnosed by imaging, or lymph node metastases pathologically confirmed after biopsy. Of the 215 cT_1-2_N_+_ patients in the study cohort, 138 (64.2%) were pathologically confirmed as having lymph node metastases, either by biopsy before NAC or by intraoperative sentinel lymph node biopsy or axillary lymph node dissection. TNM staging was performed in accordance with American Joint Committee on Cancer guidelines (version 8, 2017). Estrogen receptor (ER) and progesterone receptor (PR) status were evaluated by immunohistochemistry (IHC), with cutoffs of 1% to dichotomize tumors as positive or negative (9). Human epidermal receptor 2 (HER2) status was evaluated by IHC, with tumors having IHC scores of 2+, defined as indeterminate, further analyzed by IHC and fluorescence *in situ* hybridization (FISH) (10).

### Treatment

**Figure 1:**
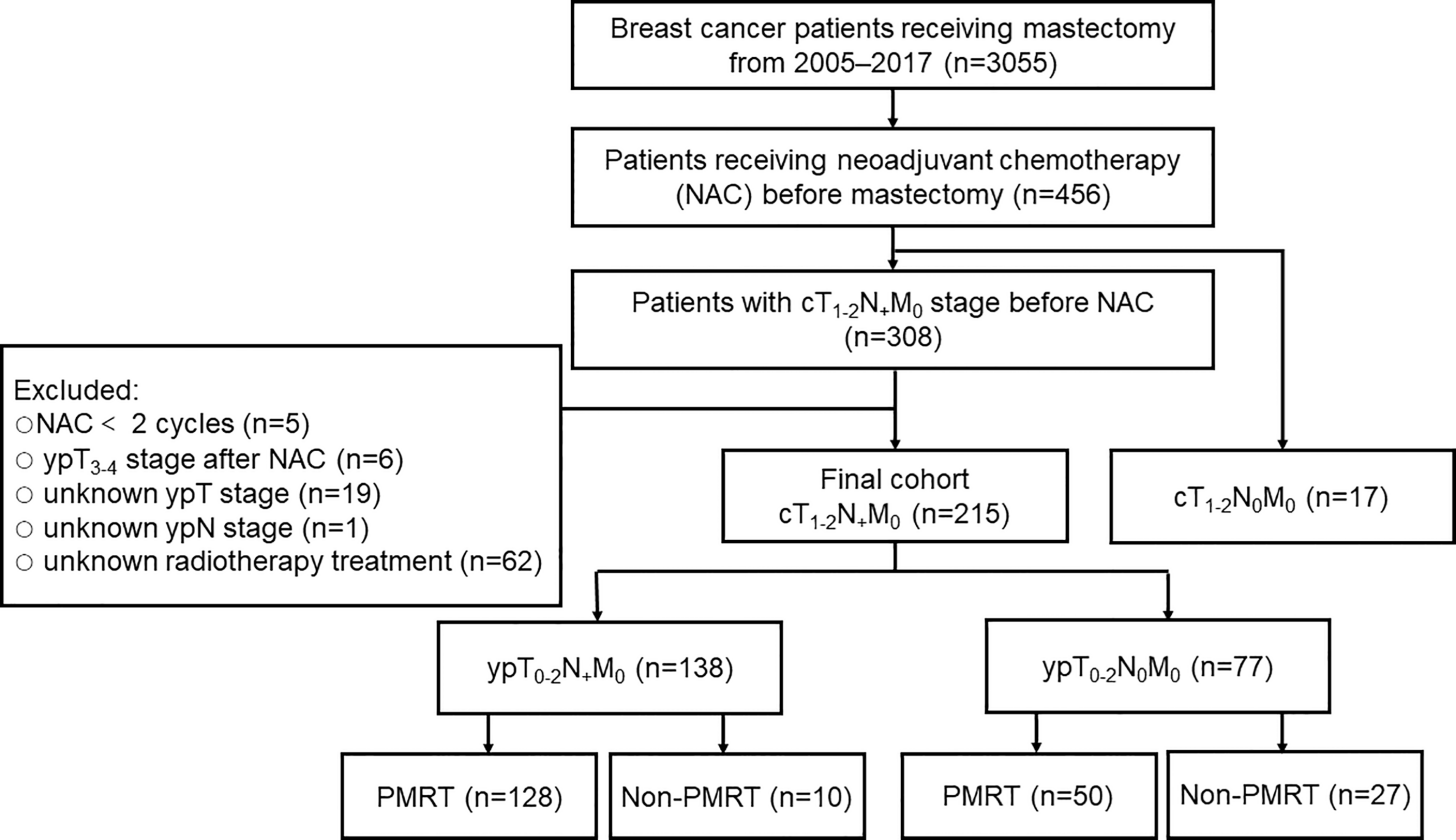
Study design. NAC, neoadjuvant chemotherapy; PMRT, postmastectomy radiotherapy; cT, clinical tumor size; cN, clinical lymph node; ypT, pathologic tumor size after neoadjuvant therapy; ypN, pathologic lymph node after neoadjuvant therapy.

The patients with cT_1-2_N_+_ breast cancer were divided into two groups, the ypT_0-2_N_+_ and the ypT_0-2_N_0_ groups, based on the pathological lymph node status of surgical specimens removed after NAC. Each of these groups was divided into two subgroups, those who did and did not receive PMRT (**Figure 1**). Of the patients in this study, 97.2% underwent axillary lymph node dissection. All hormone receptor-positive patients received adjuvant endocrine therapy, and 47 (49.5%) of the 95 patients with HER2-positive breast cancer were treated with Trastuzumab.

In patients undergoing PMRT, 50 Gy (range 45–50 Gy) radiation in 25 fractions (range 25–28) of 2 Gy each was delivered to the chest wall and/or regional lymph nodes (i.e., the supraclavicular/infraclavicular and/or internal mammary lymph node regions). Of the patients who received PMRT, 86.4% received intensity-modulated radiation therapy and 13.6% received 3D conformal radiation therapy. The chest wall and supraclavicular/infraclavicular nodal regions were generally irradiated with photons, and the internal mammary lymph node regions were irradiated with electrons.

### Study Endpoints

Patients were followed-up until September 16, 2021, with the median follow-up time being 72.6 months (5.96 years). The primary endpoint was disease-free survival (DFS), defined as the time from the date of diagnosis to the date of first locoregional recurrence (LR), distant metastasis (DM), new primary tumors in the breast, death from any cause, or last follow-up (11). The secondary endpoint was overall survival (OS), defined as the time from the date of diagnosis to the date of death due from any cause or last follow-up (11). LR included clinical, radiographic, or pathological evidence of recurrence in the ipsilateral chest wall and/or regional lymph nodes, whereas DM included recurrences at all other sites (i.e., contralateral breast, bone, lung, liver, or brain).

### Statistical Analysis

Categorical variables in two groups of patients were compared using the Pearson’s χ^2^ test or Fisher’s exact test, as appropriate, whereas continuous variables were compared by t-test. Survival, including DFS and OS, was analyzed by the Kaplan–Meier method, with differences compared by log-rank tests. Patients who were lost to follow-up were censored at the last contact. Factors associated with survival in NAC patients with residual nodal disease were assessed by univariate and multivariate Cox proportional hazards analyses, with variables found to be significant (*P*<0.10) on univariate analysis were included in the multivariate model. ER status and triple-negative breast cancer were excluded from the multivariate model due to their collinearity with molecular subtypes. All tests were two-sided, with p values < 0.05 considered statistically significant. Statistical analyses were performed using SPSS version 20.0 software (IBM Institute, Chicago, IL, USA) and GraphPad Prism version 8 software (GraphPad Inc, San Diego, CA, USA).

## Results

### Patient and Tumor Characteristics

The clinical characteristics of the 215 patients with cT_1-2_N_+_ breast cancer included in this study are illustrated in **Table 1**. Median age at diagnosis was 51.2 years (range: 25–75 years), with 188 (87.4%) of these patients diagnosed with invasive ductal carcinomas. Of these 215 patients, 46 (21.4%) and 169 (78.6%) were in clinical T_1_ and T_2_ stages, respectively. After NAC followed by mastectomy, the primary tumor staging was ypT_0_ in 40 (18.6%) patients, ypT_1_ in 103 (47.9%), and ypT_2_ in 72 (33.5%), with 77 (35.8%), 65 (30.2%), and 73 (34.0%) having lymph node stages ypN_0_, ypN_1_, and ypN_2-3_, respectively. Thirty-two (14.9%) patients had triple negative breast cancer (TNBC), and 130 (60.5%) were ER-positive, with all of the latter receiving endocrine therapy. HER2 was positive in 95 (44.1%) of patients, with 47 (49.5%) of the latter being treated with trastuzumab. Anthracycline-containing chemotherapy regimens were administered to 196 (91.2%) patients, taxane-containing regimens to 170 (79.1%), and combined anthracycline and taxane regimens to 151 (70.2%). PMRT was administered to 178 (82.8%) patients; of the latter, eight (4.5%) received irradiation to chest wall alone, 133 (74.5%) received irradiation to both the chest wall and supraclavicular/infraclavicular lymph node regions; and two (1.1%) received irradiation to chest wall, supraclavicular/infraclavicular and internal mammary lymph node regions, with the irradiation fields not specified for the remaining 35 (19.7%) patients who received PMRT.

**Table 1:** Clinical characteristics of all patients.

Variable	All patients (cT_1-2_N_+_M_0_)	
	n = 215	%
Age		
Mean	51.3	
Range	25-75	
<40	20	9.3
≥40	195	90.7
Clinical T stage		
1	46	21.4
2	169	78.6
ypT stage		
0-is	40	18.6
1	103	47.9
2	72	33.5
ypN stage		
0	77	35.8
1	65	30.2
2-3	73	34.0
Estrogen receptor status		
Positive	130	60.5
Negative	77	35.8
Unknown	8	3.7
HER2 status		
Positive	81	37.7
Negative	115	53.5
Unknown	19	8.8
TNBC		
Yes	32	14.9
No	174	80.9
Unknown	9	4.2
Molecular subtype		
Luminal A	39	18.1
Luminal B	79	36.7
HER2 overexpression	43	20.0
Triple-negative	32	14.9
Unknown	22	10.2
pCR		
Yes	25	11.6
No	190	88.4
Preoperative chemotherapy regimens		
Anthracycline containing	196	91.2
Taxane containing	170	79.1
Anthracycline and taxane containing	151	70.2
Hormone therapy/Estrogen receptor status		
	130/130	100.0
HER2-targeted therapy/HER2 status		
	44/81	54.3
PMRT		
Yes	178	82.8
No	37	17.2

cT, clinical tumor size; cN, clinical lymph node; ypT, pathologic tumor size after neoadjuvant therapy; ypN, pathologic lymph node after neoadjuvant therapy; HER2, human epidermal receptor 2; TNBC, triple negative breast cancer; pCR, pathological complete response; PMRT, postmastectomy radiotherapy.

### Requirement for PMRT After NAC in Patients With cT_1-2_N_+_ Disease Who Achieve ypT_0-2_N_0_

After NAC, 77 (35.8%) of the 215 cT_1-2_N_+_ patients achieved ypT_0-2_N_0_. Of these 77 patients, 50 (64.9%) received PMRT, whereas 27 (35.1%) did not. A comparison of clinical and treatment characteristics in these two subgroups found that only the rates of pathological complete response (pCR; 42% vs. 14.8%, *p*=0.021) and trastuzumab treatment of HER2+ patients (72.0% vs. 18.2%, *p*=0.009) differed significantly (**Table 2**).

**Table 2:** Clinical characteristics of patients in the ypT_0-2_N_0_ PMRT and non-PMRT subgroups.

Variable	ypT_0-2_N_+_				*P* value	ypT_0-2_N_0_				*P* value
	PMRT		non-PMRT			PMRT		non-PMRT		
	n = 128	%	n = 10	%		n = 50	%	n = 27	%	
Age										
Mean	51.3		53.1			51.4		50.4		
Range	30-75		39-69		1.000	25-73		33-65		0.996
<40	13	10.2	1	10.0		7	14.0	3	11.1	
≥40	115	89.8	9	90.0		43	86.0	24	88.9	
Clinical T stage					0.157					0.383
1	32	25.0	0	0.0		11	22.0	3	11.1	
2	96	75.0	10	100.0		39	78.0	24	88.9	
ypT stage					0.061					0.052
0-is	14	10.9	1	10.0		21	42.0	4	14.8	
1	68	53.1	2	20.0		18	36.0	14	51.9	
2	46	35.9	7	70.0		11	22.0	9	33.3	
ypN stage					0.426					–
0	0	0.0	0	0.0		50	100.0	27	100.0	
1	62	48.4	3	30.0		0	0.0	0	0.0	
2-3	66	51.6	7	70.0		0	0.0	0	0.0	
Estrogen receptor status					0.627					0.550
Positive	92	71.9	6	60.0		20	40.0	10	37.0	
Negative	31	24.2	4	40.0		28	56.0	14	51.9	
Unknown	5	3.9	0	0.0		2	4.0	3	11.1	
HER2 status					0.889					0.158
Positive	40	31.3	4	40.0		25	50.0	11	40.7	
Negative	72	56.3	5	50.0		25	50.0	14	51.9	
Unknown	14	10.9	1	10.0		0	0.0	2	7.4	
TNBC					0.704					0.267
Yes	12	9.4	0	0.0		13	26.0	7	25.9	
No	112	87.5	10	100.0		36	72.0	17	63.0	
Unknown	4	3.1	0	0.0		1	2.0	3	11.1	
Molecular subtype					0.364					0.518
Luminal A	29	22.7	2	20.0		4	8.0	4	14.8	
Luminal B	56	43.8	3	30.0		15	30.0	5	18.5	
HER2 overexpression	22	17.2	3	30.0		15	30.0	7	25.9	
Triple-negative	12	9.4	0	0.0		13	26.0	7	25.9	
Unknown	9	7.0	2	20.0		3	6.0	4	14.8	
pCR					–					**0.021**
Yes	0	0.0	0	0.0		21	42.0	4	14.8	
No	128	100.0	10	100.0		29	58.0	23	85.2	
Preoperative chemotherapy regimens					0.964					0.172
Anthracycline containing	118	92.2	10	100.0		41	82.0	27	100.0	
Taxane containing	99	77.3	7	70.0		48	96.0	16	59.3	
Anthracycline and taxane containing	89	69.5	7	70.0		39	78.0	16	59.3	
Hormone therapy/Estrogen receptor status					–					–
	92/92	100.0	6/6	100.0		20/20	100.0	10/10	100.0	
HER2-targeted therapy/HER2 status					1.000					**0.009**
	21/40	50.0	3/5	60.0		18/25	72.0	2/11	18.2	

ypT, pathologic tumor size after neoadjuvant therapy; ypN, pathologic lymph node after neoadjuvant therapy; PMRT, postmastectomy radiotherapy; HER2, human epidermal receptor 2; TNBC, triple negative breast cancer; pCR, pathological complete response. P value in bold indicates statistically significant.

Eleven (22%) of the 50 patients in the ypT_0-2_N_0_ PMRT subgroup and seven (25.9%) of the 27 patients in the ypT_0-2_N_0_ non-PMRT subgroup had LR or DM. Analysis of the recurrence patterns in these subgroups showed that the LR rate was significantly lower in the ypT_0-2_N_0_ PMRT than in the ypT_0-2_N_0_ non-PMRT subgroup (2% vs. 14.8%, *p*=0.048), whereas the DM rate did not differ in these two subgroups (**Table 3**).

**Table 3:** Recurrence patterns in the ypT_0-2_N_0_ PMRT and non-PMRT subgroups.

Initial recurrent sites	ypT_0-2_N_0_M_0_		
	PMRT	non-PMRT	*P value*
	(n = 50)	(n = 27)	
Locoregional*	1 (2%)	4 (14.8%)	**0.048**
Distant metastasis	10 (20%)	3 (11.1%)	0.500

*Represents the patient who had chest wall, supraclavicular, or axillary LN recurrence. PMRT, postmastectomy radiotherapy; ypT, pathologic tumor size after neoadjuvant therapy; ypN, pathologic lymph node after neoadjuvant therapy. P value in bold indicates statistically significant.

**Figure 2:**
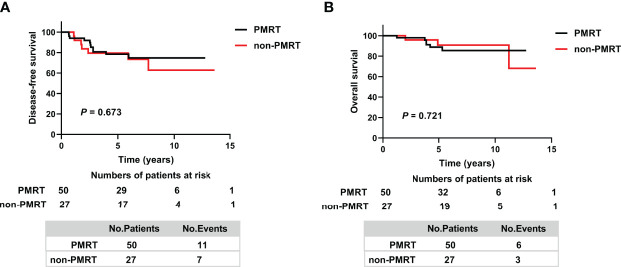
Kaplan–Meier analysis of **(A)** Disease-free survival and **(B)** Overall survival in patients who achieved ypT_0-2_N_0_ after NAC and in the PMRT and non-PMRT subgroups. PMRT, postmastectomy radiotherapy.

The 5-year DFS rates were similar in the ypT_0-2_N_0_ PMRT and ypT_0-2_N_0_ non-PMRT subgroups (78.5% vs. 79.5%, *p* = 0.673) (**Figure 2A**). Similarly, the 5-year OS rate was 88.8% (95% CI 79.6–98.0%) in the ypT_0-2_N_0_ PMRT subgroup and 90.8% (95% CI 78.6–103%) in the ypT_0-2_N_0_ non-PMRT subgroup, a difference that was not statistically significant (*p* = 0.721) (**Figure 2B**).

### Comparative Prognoses of cT_1-2_N_+_ Patients Who Achieve ypT_0-2_N_0_ Without PMRT and cT_1-2_N_0_ Patients?

**Figure 3:**
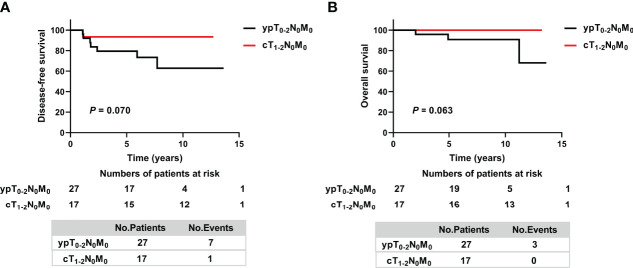
Kaplan–Meier analysis of **(A)** Disease-free survival and **(B)** Overall survival in cT_1-2_N_+_ patients who achieved ypT_0-2_N_0_ after NAC without PMRT and cT_1-2_N_0_ non-PMRT patients. ypT, pathologic tumor size after neoadjuvant therapy; ypN, pathologic lymph node after neoadjuvant therapy; cT, clinical tumor size; cN, clinical lymph node.

Because the prognosis of patients in the ypT_0-2_N_0_ non-PMRT subgroup was non-inferior to that of patients in the ypT_0-2_N_0_ PMRT subgroup, the survival of ypT_0-2_N_0_ non-PMRT patients was compared with that of patients with cT_1-2_N_0_ stage tumors before NAC. The clinical and treatment characteristics of these two groups did not differ significantly (**Supplementary Table 1**), nor did their 5-year DFS rates (79.5% vs. 93.3%, *p* = 0.070) (**Figure 3A**). By the date of the last follow-up, no patient in the cT_1-2_N_0_ group had died. The 5-year OS rates in the ypT_0-2_N_0_ non-PMRT and cT_1-2_N_0_ group also did not differ significantly (*p* = 0.063) (**Figure 3B**).

### Correlations Between Clinical Variables and Prognosis in NAC Patients With Residual Nodal Disease

Based on National Cancer Institute guidelines (12), 128 (92.8%) of the 138 patients with residual nodal disease after NAC and surgery (ypT_0-2_N_+_) received PMRT. Analysis of the correlations between clinical variables and prognosis in these patients showed that various prognostic factors correlated with DFS and OS (**Table 4**). Univariate analyses showed that ypN stage, ER status and molecular subtypes, including HER2 overexpression and TNBC were significantly associated with both DFS and OS. Distant recurrence and all-cause mortality rates were was 1.98 and 4.19 times higher, respectively, in patients with ypN_2-3_ than in those with ypN_1_. Multivariate analyses showed that HER2 overexpression and TNBC were significant predictors of poorer DFS and OS, whereas ypN stage was a significant independent predictor of OS in the ypT_0-2_N_+_ PMRT subgroup (**Table 5**).

**Table 4:** Univariate analysis of factors associated with DFS and OS in the ypT_0-2_N_+_ PMRT subgroup.

Variable		DFS		OS	
	No. of patients	HR (95% CI)	*P* value	HR (95% CI)	*P* value
Age			0.973		0.739
<40	13	ref.		ref.	
≥40	115	1.020 (0.313-3.33)		1.410 (0.187-10.62)	
Clinical T stage			0.380		0.125
1	32	ref.		ref.	
2	96	1.420 (0.649-3.108)		3.160 (0.726-13.756)	
ypT stage			0.174		0.168
0-is	14	ref.		ref.	
1	68	0.666 (0.348-1.272)		0.681 (0.288-1.608)	
2	46	1.550 (0.905-2.655)		2.001 (0.918-4.36)	
ypN stage			**0.043**		**0.011**
1	62	ref.		ref.	
2-3	66	1.981 (1.023-3.837)		4.189 (1.387-12.648)	
Estrogen receptor status			**0.001**		**0.010**
Positive	92	ref.		ref.	
Negative	31	3.037 (1.572-5.867)		3.329 (1.337-8.29)	
HER2 status			0.166		0.194
Positive	40	ref.		ref.	
Negative	72	0.613 (0.307-1.224)		0.532 (0.205-1.379)	
TNBC			**0.024**		**0.041**
Yes	12	ref.		ref.	
No	112	0.387 (0.17-0.882)		0.313 (0.103-0.955)	
Molecular subtype			**0.020**		**0.030**
Luminal A	28	ref.		ref.	
Luminal B	55	2.114 (0.706-6.324)		4.865 (0.613-38.639)	
HER2 overexpression	10	4.944 (1.486-16.451)		10.077 (1.114-91.159)	
Triple-negative	17	4.648 (1.310-16.486)		12.693 (1.412-114.062)	
Preoperative chemotherapy regimens					
			0.238		0.078
Anthracycline containing	118	ref.		ref.	
Without anthracycline	11	1.780 (0.683-4.642)		2.797 (0.892-8.771)	
			0.582		0.807
Taxane containing	99	ref.		ref.	
Without Taxane	30	1.226 (0.594-2.530)		0.879 (0.290-2.662)	
			0.23		0.371
Anthracycline and Taxane containing	89	ref.		ref.	
Without both anthracycline and Taxane	40	1.497 (0.775-2.893)		1.547 (0.608-3.933)	

ypT, pathologic tumor size after neoadjuvant therapy; ypN, pathologic lymph node after neoadjuvant therapy; PMRT, postmastectomy radiotherapy; DFS, disease-free survival; OS, overall survival; HR, hazard ratio; CI, confidence interval; ref reference; HER2, human epidermal receptor 2; TNBC, triple negative breast cancer; pCR, pathological complete response. P value in bold indicates statistically significant.

**Table 5:** Multivariate analysis of factors associated with DFS and OS in the ypT_0-2_N_+_ PMRT subgroup.

Variable		DFS		OS	
	No. of patients	HR (95% CI)	*P* value	HR (95% CI)	*P* value
ypN stage			0.222		**0.024**
1	55	ref.		ref.	
2-3	60	1.549 (0.767-3.127)		3.687 (1.184-11.480)	
Molecular subtype			**0.022**		**0.039**
Luminal A	29	ref.		ref.	
Luminal B	56	2.021 (0.674-6.060)		4.512 (0.566-35.982)	
HER2 overexpression	12	4.167 (1.162-14.947)		9.709 (1.066-88.391)	
Triple-negative	18	5.138 (1.541-17.130)		11.402 (1.255-103.612)	

DFS, disease-free survival; OS, overall survival; PMRT, postmastectomy radiotherapy; HR, hazard ratio; CI, confidence interval; ypT, pathologic tumor size after neoadjuvant therapy; ypN, pathologic lymph node after neoadjuvant therapy; HER2, human epidermal receptor 2. P value in bold indicates statistically significant.

### Indications for PMRT After NAC: Current Literature Review

To further assess whether PMRT can benefit patients after NAC, the PubMed database was systematically searched for using the search terms, “postmastectomy radiation therapy”, “neoadjuvant chemotherapy” and “breast cancer”, for studies of PMRT after NAC published from March 1993 to November 2021. Of the 184 articles identified, nine (13–21) report results similar to ours (**Table 6**). Eight of these studies reported the results of event-free survival analysis, such as locoregional recurrence (LRR), local recurrence free survival (LRFS), DFS, recurrence-free survival (RFS), and distant metastasis-free survival (DMFS) (13–16, 18–21), whereas six studies reported OS and cause-specific survival (CSS) rates (13–17, 20).

**Table 6:** Previous studies analyzing the effects of PMRT after NAC.

Study	Luo et al.	Huang et al. (13)	McGuire et al. (14)	Le Scodan et al. (15)	Shim et al. (16)	Rusthoven et al. (17)	Cao et al. (18)	Wang et al. (19)	Wang et al. (20)	Zhang et al. (21)
(year)	(2022)	(2004)	(2007)	(2012)	(2014)	(2016)	(2017)	(2018)	(2020)	(2021)
Follow-up (years)	6.0	5.8	5.2	7.6	4.9	3.25	5.6	5.1	6.0	5.4
No. of cases	215	676	106	134	151	10283	88	217	142	554
Mean ages (years)	51.2	48-49	NA	50	47	NA	48	50	49	51
Clinical T stage	cT_1-2_ (100%)	cT_1-2_ (21.4%) cT_3-4_ (78.6%)	cT_1-2_ (33%) cT_3-4_ (67%)	cT_1-2_ (50.7%) cT_3-4_ (49.3%)	cT_1-2_ (49.0%) cT_3-4_ (51.0%)	cT_1-2_ (59.9%) cT_3_ (40.1%)	cT_1-2_ (100%)	cT_1-2_ (100%)	cT_1-2_ (100%)	cT_1-2_ (79.1%) cT_3-4_ (20.9%)
Clinical N stage	cN_+_ (100%)	cN_+_ (79.4%)	cN_+_(71.7%)	cN_+_(47.8%)	cN_+_(84.8%)	cN_+_ (100%)	cN_+_ (100%)	cN_+_ (75.6%)	cN_+_ (100%)	cN_+_ (76.5%)
ypT stage	ypT_0-2_(100%)	ypT_0-2_ (86.1%) ypT_3-4_ (12.6%)	ypT_0_ (100%)	NA	ypT_0-1_ (62.9%) ypT_2-4_ (37.1%)	NA	ypT_0-2_ (93.2%) ypT_3-4_ (2.2%)	ypT_0-2_ (92.2%) ypT_3-4_ (7.8%)	ypT_1-2_ (100%)	ypT_0-1_ (60.3%) ypT_2-4_ (39.7%)
ypN stage	ypN_0_ (34.9%) ypN_+_ (64.3%)	ypN_0_ (29.7%) ypN_+_ (68.9%)	ypN_0_ (100%)	ypN_0_ (100%)	ypN_0_ (100%)	ypN_0_ (29.6%) ypN_+_ (70.4%)	ypN_0_ (60.2%) ypN_+_ (39.8%)	ypN_0_ (26.7%) ypN_+_ (73.3%)	ypN_0_ (100%)	ypN_0_ (31%)
pCR	pCR(10.4%)	pCR(12.7%)	pCR (100%)	pCR (17.9%)	NA	pCR(16.3%)	pCR(27.3%)	NA	pCR(33.8%)	pCR (6.9%)
PMRT	PMRT(82.8%)	PMRT(80.2%)	PMRT(67.9%)	PMRT(58.2%)	PMRT(69.5%)	PMRT(71.8%)	PMRT(85.2%)	PMRT(59.0%)	PMRT(77.5%)	PMRT (72.0%)
NAC regimens	A containing(91.2%) T containing(79.1%) A and T containing (70.2%)	NA	A containing (92%) T containing (38%)	A-based (90.3%) T-based (9.7%)	A-based (36.4%) T-based (6%) A and T (55.6%)	NA	A-based (25%) T-based (30.7%) A and T (5.7%)	NA	A-based (2.1%) T-based (15.5%) A and T (82.4%)	A and T (75%)
LRR/LRFS	NA	10-yr LRR: PMRT vs non-PMRT in cT_1_: 8% vs 0% (*P*>0.050); in cT_2_: 10% vs 7% (*P*>0.050)	10-yr LRR: PMRT vs non-PMRT in clinical stage I or II: 0% vs 0% (*P*>0.050); in stage III: 7.3% vs 33.3% **(*P*=0.04)**	10-yr LRFS: PMRT vs non-PMRT: 96.2% vs 86.8% (*P*>0.050)	10-yr LRFS: PMRT vs non-PMRT: 98.1% vs 92.3% (*P*>0.050)	NA	5-yr LRFS: PMRT vs non-PMRT: 96.9% vs 78.6% **(*P*=0.020)**	5-yr LRR: PMRT vs non-PMRT in low-risk group: 3.3% vs 1.7% (*P*>0.050); in high-risk group: 21.8% vs 42.2% **(*P*=0.031)**	5-yr LRFS: PMRT vs non-PMRT: 94.5% vs 90.1% (*P*>0.050)	5-yr LRR: PMRT vs non-PMRT: 7.3% vs 14.1% **(*P*=0.01)**
DFS/RFS/DMFS	5-yr DFS: PMRT vs non-PMRT in ypT_0-2_N_0_: 74.7% vs 73.3% (*P*>0.050)	NA	10-yr DMFS: PMRT vs non-PMRT in stage III: 40.7% vs 87.9% **(*P<*0.01)**	NA	10-yr DFS: PMRT vs non-PMRT: 91.2% vs 83% (*P*>0.050)	NA	5-yr DMFS: PMRT vs non-PMRT: 92.9% vs 81.5% (*P*>0.050) 5-yr DFS: PMRT vs non-PMRT: 92.9% vs 72.9% (*P*>0.050)	NA	5-yr RFS: PMRT vs non-PMRT: 88.7% vs 72.4%**(*P*=0.028)**	5-yr DFS: PMRT vs non-PMRT: 74% vs 74.8% (*P*>0.050)
OS/CSS	5-yr OS: PMRT vs non-PMRT in ypT_0-2_N_0_: 85.5% vs 90.8% (*P*>0.050)	10-yr CSS: PMRT vs non-PMRT in cT_1_: 92% vs 80% (*P*>0.050); in cT_2_: 66% vs 56% (*P*>0.050)	10-yr OS: PMRT vs non-PMRT in stage III: 77.3% vs 33.3% **(*P*<0.01)**	10-yr OS: PMRT vs non-PMRT: 77.2% vs 87.7% (*P*>0.050)	10-yr OS: PMRT vs non-PMRT: 93.3% vs 89.9% (*P*>0.050)	5-yr OS: PMRT vs non-PMRT in ypN_0_: 88.3% vs 84.8% **(*P*=0.019)**; in ypN_+_: 74.1% vs 70.9% **(*P* < 0.010)**	NA	NA	5-yr OS: PMRT vs non-PMRT: 96.1% vs 95% (*P*>0.050)	NA
Conclusion	PMRT didn’t improve 5-yr DFS and 5-yr OS in cT_1-2_N_+_ breast cancer patients with ypT_0-2_N_0_ after NAC.	PMRT didn’t decrease 10-yr LRR and didn’t improve 10-yr CSS in cT_1-2_ patients after NAC.	PMRT didn’t improve 10-yr LRR in clinical stage I-II patients with pCR after NAC, but significantly improve 10-yr LRR, DMFS and OS in those of clinical stage III patients.	PMRT didn’t improve 10-yr LRFS and OS in clinical stage II-III patients with pN_0_ after NAC.	PMRT didn’t improve 10-yr LRFS, DFS and OS in clinical stage II-III breast cancer patients with pN_0_ after NAC	PMRT significantly improved 5-yr OS in cT_1-3_N_1_ patients after NAC, whatever achieving ypN_0_ or still remaining ypN+ patients.	PMRT significantly improved 5-yr LRFS in cT_1-2_N_1_ patients after NAC, but didn’t affect 5-yr DMFS and DFS. OS is not evaluated.	PMRT didin’t decrease 5-yr LRR in cT_1-2_N_0-1_ patients with low risk, but significantly decrease 5-yr LRR in those with high risk (risk factors including ypN stage, histologic grade and LVI).	PMRT significantly improved 5-yr RFS in cT_1-2_N_1_ patients who achieving ypT_1-2_N_0_ after NAC, but didn’t improve 5-yr LRFS and OS.	PMRT significantly reduced 5-yr LRR in clinical stage II-III patients after NAC, among whom with ypN_0_ derived no local control or survival benefit from PMRT.

PMRT, postmastectomy radiotherapy; NAC, neoadjuvant chemotherapy; NA, not applicable; cT, clinical tumor size; cN, clinical lymph node; ypT, pathologic tumor size after neoadjuvant therapy; ypN, pathologic lymph node after neoadjuvant therapy; pCR, pathological complete response; A, anthracycline; T, Taxane; 5-yr 5-year; 10-yr 10-year; LRR, local regional recurrence; LRFS, local recurrence free survival; DFS, disease-free survival; RFS, recurrence-free survival; DMFS, distant metastases-free survival; OS, overall survival; CSS, cause-specific survival. P value in bold indicates statistically significant.

## Discussion

Both prospective and retrospective studies have provided evidence for recommending PMRT after NAC for breast cancer patients with cT_3-4_, cN_2-3_ or residual lymph node disease, as well as omitting PMRT for cT_1-2_N_0-1_ patients who develop a pCR (22). However, the benefits of PMRT after NAC in breast cancer patients with initial clinical stage cT_1-2_N_+_, especially for those who achieved ypT_1-2_N_0_, remain largely unknown. The present study retrospectively analyzed outcomes of PMRT after NAC in cT_1-2_N_+_ breast cancer patients in our medical center. These findings suggest that an effective response to NAC (ypT_0-2_N_0_) is indicative of a sufficiently favorable prognosis and that PMRT may be unnecessary for cT_1-2_N_+_ patients who achieve ypT_0-2_N_0_ after NAC.

Most of the clinical and treatment characteristics of patients in the ypT_0-2_N_0_ PMRT and ypT_0-2_N_0_ non-PMRT groups did not differ significantly, except for pCR rate and the percentages of HER2+ patients treated with trastuzumab. The pCR rate was lower in patients in the ypT_0-2_N_0_ non-PMRT group, which may have been due to the lower percentage of patients in this group who had completed NAC regimens (33.3% vs. 64%, *p* = 0.010) (**Supplementary Table 2**). The inclusion of patients who had received adjuvant chemotherapy in this analysis resulted in similar percentages of patients in the ypT_0-2_N_0_ PMRT and ypT_0-2_N_0_ PMRT non-PMRT groups who had completed both neoadjuvant and adjuvant chemotherapy regimens (96% vs. 92.6%, *p* = 0.609). The findings in this study suggest that the chemotherapy completion rate was more related to patient prognosis than the completion of NAC alone.

Of the HER2+ patients in the ypT_0-2_N_0_ group, only 55.6% were treated with trastuzumab, perhaps because this agent was not approved for local medical insurance until September 2017. The percentage of HER2+ patients receiving trastuzumab was lower in the ypT_0-2_N_0_ non-PMRT than in the ypT_0-2_N_0_ PMRT group, resulting in inadequate treatment of the former. Even under all these circumstances, however, the 5-year DFS and OS rates did not differ significantly in these two subgroups, providing further evidence that ypT_0-2_N_0_ patients have a favorable prognosis, even without PMRT.

Interestingly survival outcomes did not differ significantly in cT_1-2_N_+_ patients who achieved ypT_0-2_N_0_ without PMRT and cT_1-2_N_0_ patients before NAC. Although there were concerns that the lymph node status of cT_1-2_N_0_ might be downstaged, all the cT_1-2_N_0_ patients remained ypT_1-2_N_0_ after NAC. Based on PMRT guidelines, T_1-2_N_0_ stage patients without NAC do not need PMRT (6, 7). None of the patients in the present study with cT_1-2_N_0_ received PMRT after NAC, while having favorable 5-year DFS and OS rates. Thus, the comparable survival of cT_1-2_N_+_ patients who achieved ypT_0-2_N_0_ without PMRT and cT_1-2_N_0_ patients suggested the former can show favorable survival outcomes, even in the absence of PMRT. However, due to the limited sample size in ypT_0-2_N_0_ and cT_0-2_N_0_ subgroup, there is a possibility of increasing type I error caused by limited sample size.

Two ongoing prospective trials are addressing the need for PMRT in cT_1-2_N_+_ patients who achieve nodal pCR after NAC. The NSABP51 trial is a randomized phase III clinical trial evaluating PMRT in pathologically proven cT_1-3_N_1_ patients who convert to pN_0_ after NAC (www.nsabp.pitt.edu/B-51.asp). The RAPCHEM trial is prospective observational study evaluating the 5-year LRR rate in cT_1-2_N_0-1_ patients after NAC, breast surgery and radiotherapy, with protocols based on ypTNM stage (https://clinicaltrials.gov/ct2/show/study/NCT01279304). The protocol of the RAPCHEM trial resulted in the stratification of patients with ypN_0_ to a low risk group, with these patients not receiving PMRT.

Several previous retrospective studies have analyzed the need for PMRT in subjects who received NAC. For example, a study of 676 patients with locally advanced breast cancer, including 145 with cT_1-2_ stage tumors, who were treated with NAC and mastectomy, found that PMRT did not reduce LRR or improve 10-years CSS in patients with cT_1-2_ stage tumors (13). Although only 29.7% of patients achieved ypN_0_ and 68.9% had residual nodal disease after NAC, PMRT did not benefit survival. Another study of 106 cT_1-4_N_+_ breast cancer patients who achieved pCR after NAC also found that PMRT did not improve 10-year LRR in clinical stage I-II patients, but significantly improved 10-year LRR, DMFS and OS in clinical stage III patients (14), suggesting that PMRT may be more likely to improve survival in patients with more advanced stage tumors. Similarly, two other studies found that PMRT did not improve 10-year LRFS and OS in clinical stage II-III patients who achieved pN_0_ after NAC (15, 16). A large study in 10,283 cN_+_ patients found that PMRT significantly improved 5-year OS in cT_1-3_N_1_ patients after NAC, whether they achieved ypN_0_ or remained ypN_+_ (17). However, 40.1% of these patients had stage cT_3_ at diagnosis, a class of patients shown to benefit from PMRT, with clinical consensus indicating that PMRT after NAC can improve survival in patients with clinical stage III breast cancer (i.e., T_3_N_1_) (12). A study of 88 cT_1-2_N_+_ patients who developed ypN_0_ after NAC found that PMRT significantly improved 5-year LRFS, but had no effect on DMFS and DFS (18). Because 39.8% of these patients remained ypN_1_, a class that can gain survival benefits from PMRT, the effect of PMRT on cT_1-2_N_+_ patients who achieve ypN_0_ after NAC has not yet been clarified. Interestingly, a study of 217 cT_1-2_N_0-1_ patients found that PMRT significantly reduced 5-year LRR in high risk, but not in low risk, patients (19). Risk factors in that study included ypN stage, histologic grade and lymphatic vessel invasion. The cT_1-2_N_+_ patients who achieved ypN_0_ after NAC in our study may comparable to the low risk cT_1-2_N_0-1_ population in the previous study (19), in which PMRT did not decrease 5-year LRR. An analysis of 142 cT_1-2_N_1_ breast cancer patients found that PMRT did not improve OS in cT_1-2_N_1_ patients who achieved ypT_1-2_N_0_ after NAC, results consistent with the findings of the present study (20). That study, however, found that PMRT significantly improved RFS but not LRFS, whereas rates of locoregional recurrence and distant metastases did not difference in patients who did and did not receive PMRT. A recent study of 554 clinical stage II-III patients found that PMRT after NAC significantly reduced 5-year LRR in clinical stage II-III patients, but had no local control or survival benefit in patients with ypN_0_ after NAC (21).

To date, no Grade 1 evidence has shown that PMRT can be omitted in cT_1-2_N_+_ patients who achieve ypT_0-2_N_0_ after NAC. Although the present study suggests that PMRT can be omitted, this study had several limitations. First, this was a retrospective study, with selection bias between the PMRT and non-PMRT groups being the most important inherent shortcoming. Secondly, the sample size and number of events in this study are limited, which may lead to the statistical type I error. With interest, we will continue to follow up the outcomes of ypT_0-2_N_0_ PMRT and non-PMRT patients in our hospital, and may conduct a prospective study to investigate the possibility of exempting radiotherapy in cT_1-2_N_+_ breast cancer patients with ypT_0-2_N_0_ after NAC. Thirdly, data collection was subject to information bias due to the loss of individual data and limited follow-up time. Fourthly, the percentage of HER2+ patients treated with trastuzumab was low, resulting in inadequate treatment. Fifthly, incomplete neoadjuvant chemotherapy in ypT_0-2_N_0_ non-PMRT subgroup may cause lower lymph node complete response rate (ypN_0_), which might lead to fewer patients enrolled in ypT_1-2_N_0_ group. In addition, initial axillary lymph node status in a small proportion of patients (16.3%) was not determined by pathology.

In conclusion, this study found that PMRT did not affect survival in cT_1-2_N_+_ breast cancer patients who achieved ypT_0-2_N_0_ after NAC. Without PMRT, the prognosis of cT_1-2_N_+_ patients who achieved ypT_0-2_N_0_ after NAC did not differ significantly from that of cT_1-2_N_0_ patients, suggesting that PMRT may be safely omitted for cT_1-2_N_+_ breast cancer patients who achieve ypT_0-2_N_0_ after NAC. Prospective studies in larger patient populations are warranted.

## Data Availability Statement

In this study, all the patients’ data were from the Second Affiliated Hospital, Zhejiang University School of Medicine, which are not publicly available in order to protect patient privacy, but can be accessed from the corresponding author, Dr. Jiaojiao Zhou (zhoujj@zju.edu.cn), on reasonable request.

## Ethics Statement

This study was approved by the Ethics Committee of our hospital (Approval No: 2020-363) with waiver of informed consent.

## Author Contributions

JZ, YC and SZ planned and designed this study. JZ, YC drew the outline of this study. JZ, ML and HC wrote the manuscript. ML, HC, HD and YJ collected the all the clinical data. ML and HC performed the data analysis. HD, YJ, KZ and HM did the follow-up of patients. ML, HC and GW participated in searching relevant literatures. All authors have read and approved the final manuscript.

## Funding

This study was supported by the National Natural Science Foundation of China (Grant No. 82172344, 81702866), the funding of the Key Program of the Natural Science Foundation of Zhejiang Province (Grant No. LZ16H160002), the funding of Medical Science and Technology Project of Zhejiang Province (Grant No. 2022RC174), the Fundamental Research Funds for the Central Universities (Grant No. 2021FZZX002-09)

## Conflict of Interest

The authors declare that the research was conducted in the absence of any commercial or financial relationships that could be construed as a potential conflict of interest.

## Publisher’s Note

All claims expressed in this article are solely those of the authors and do not necessarily represent those of their affiliated organizations, or those of the publisher, the editors and the reviewers. Any product that may be evaluated in this article, or claim that may be made by its manufacturer, is not guaranteed or endorsed by the publisher.

## Abbreviations

PMRT, postmastectomy radiotherapy; NAC, neoadjuvant chemotherapy; cT, clinical tumor size; cN, clinical lymph node; ypT, pathologic tumor size after neoadjuvant therapy; ypN, pathologic lymph node after neoadjuvant therapy; DFS, disease-free survival; OS, overall survival; CI, confidence interval; ER, estrogen receptor; PR, progesterone receptor; IHC, immunohistochemistry; HER2, human epidermal receptor 2; LR, locoregional recurrence; DM, distant metastasis; TNBC, triple negative breast cancer; pCR, pathological complete response; LRR, local regional recurrence; LRFS, local recurrence free survival; RFS, recurrence-free survival; DMFS, distant metastasesfreesurvival; CSS cause-specific survival.

## References

[1] Ikeda T Jinno H Matsu A Masamura S Kitajima M. The Role of Neoadjuvant Chemotherapy for Breast Cancer Treatment. Breast Cancer (Tokyo Japan) (2002) 9(1):8–14. doi: 10.1007/BF02967540 12196715

[2] Hayes DF Schott AF. Neoadjuvant Chemotherapy: What Are the Benefits for the Patient and for the Investigator? J Natl Cancer Inst Monogr (2015) 2015(51):36–9. doi: 10.1093/jncimonographs/lgv004 26063884

[3] Group EBCTC. Long-Term Outcomes for Neoadjuvant Versus Adjuvant Chemotherapy in Early Breast Cancer: Meta-Analysis of Individual Patient Data From Ten Randomised Trials. Lancet Oncol (2018) 19(1):27–39. doi: 10.1016/S1470-2045(17)30777-5 29242041 PMC5757427

[4] Fisher B Brown A Mamounas E Wieand S Robidoux A Margolese RG. Effect of Preoperative Chemotherapy on Local-Regional Disease in Women With Operable Breast Cancer: Findings From National Surgical Adjuvant Breast and Bowel Project B-18. J Clin Oncol (1997) 15(7):2483–93. doi: 10.1200/JCO.1997.15.7.2483 9215816

[5] Kuerer HM Sahin AA Hunt KK Newman LA Breslin TM Ames FC. Incidence and Impact of Documented Eradication of Breast Cancer Axillary Lymph Node Metastases Before Surgery in Patients Treated With Neoadjuvant Chemotherapy. Ann Surg (1999) 230(1):72–8. doi: 10.1097/00000658-199907000-00011 PMC1420847 10400039

[6] Taylor ME Haffty BG Rabinovitch R Arthur DW Halberg FE Strom EA. Acr Appropriateness Criteria on Postmastectomy Radiotherapy Expert Panel on Radiation Oncology-Breast. Int J Radiat Oncol Biol Phys (2009) 73(4):997–1002. doi: 10.1016/j.ijrobp.2008.10.080 19251087

[7] Recht A Comen EA Fine RE Fleming GF Hardenbergh PH Ho AY. Postmastectomy Radiotherapy: An American Society of Clinical Oncology, American Society for Radiation Oncology, and Society of Surgical Oncology Focused Guideline Update. J Clin Oncol (2016) 34(36):4431–42. doi: 10.1200/JCO.2016.69.1188 27646947

[8] Kronowitz SJ Robb GL. Breast Reconstruction With Postmastectomy Radiation Therapy: Current Issues. Plast Reconstr Surg (2004) 114(4):950–60. doi: 10.1097/01.prs.0000133200.99826.7f 15468404

[9] Hammond MEH Hayes DF Wolff AC Mangu PB Temin S. American Society of Clinical Oncology/College of American Pathologists Guideline Recommendations for Immunohistochemical Testing of Estrogen and Progesterone Receptors in Breast Cancer. J Oncol Pract (2010) 6(4):195–7. doi: 10.1200/JOP.777003 PMC2900870 21037871

[10] Wolff AC Hammond MEH Allison KH Harvey BE Mangu PB Bartlett JMS. Human Epidermal Growth Factor Receptor 2 Testing in Breast Cancer: American Society of Clinical Oncology/College of American Pathologists Clinical Practice Guideline Focused Update. J Clin Oncol (2018) 36(20):2105–22. doi: 10.1200/JCO.2018.77.8738 29846122

[11] Huo D Hu H Rhie SK Gamazon ER Cherniack AD Liu J. Comparison of Breast Cancer Molecular Features and Survival by African and European Ancestry in the Cancer Genome Atlas. JAMA Oncol (2017) 3(12):1654–62. doi: 10.1001/jamaoncol.2017.0595 PMC5671371 28472234

[12] Buchholz TA Lehman CD Harris JR Pockaj BA Khouri N Hylton NF. Statement of the Science Concerning Locoregional Treatments After Preoperative Chemotherapy for Breast Cancer: A National Cancer Institute Conference. J Clin Oncol (2008) 26(5):791–7. doi: 10.1200/JCO.2007.15.0326 18258988

[13] Huang EH Tucker SL Strom EA McNeese MD Kuerer HM Buzdar AU. Postmastectomy Radiation Improves Local-Regional Control and Survival for Selected Patients With Locally Advanced Breast Cancer Treated With Neoadjuvant Chemotherapy and Mastectomy. J Clin Oncol (2004) 22(23):4691–9. doi: 10.1200/JCO.2004.11.129 15570071

[14] McGuire SE Gonzalez-Angulo AM Huang EH Tucker SL Kau S-WC Yu T-K. Postmastectomy Radiation Improves the Outcome of Patients With Locally Advanced Breast Cancer Who Achieve a Pathologic Complete Response to Neoadjuvant Chemotherapy. Int J Radiat Oncol Biol Phys (2007) 68(4):1004–9. doi: 10.1016/j.ijrobp.2007.01.023 PMC4329732 17418973

[15] Le Scodan R Selz J Stevens D Bollet MA de la Lande B Daveau C. Radiotherapy for Stage Ii and Stage Iii Breast Cancer Patients With Negative Lymph Nodes After Preoperative Chemotherapy and Mastectomy. Int J Radiat Oncol Biol Phys (2012) 82(1):e1–7. doi: 10.1016/j.ijrobp.2010.12.054 21377284

[16] Shim SJ Park W Huh SJ Choi DH Shin KH Lee NK. The Role of Postmastectomy Radiation Therapy After Neoadjuvant Chemotherapy in Clinical Stage Ii-Iii Breast Cancer Patients With Pn0: A Multicenter, Retrospective Study (Krog 12-05). Int J Radiat Oncol Biol Phys (2014) 88(1):65–72. doi: 10.1016/j.ijrobp.2013.09.021 24161425

[17] Rusthoven CG Rabinovitch RA Jones BL Koshy M Amini A Yeh N. The Impact of Postmastectomy and Regional Nodal Radiation After Neoadjuvant Chemotherapy for Clinically Lymph Node-Positive Breast Cancer: A National Cancer Database (Ncdb) Analysis. Ann Oncol (2016) 27(5):818–27. doi: 10.1093/annonc/mdw046 26861597

[18] Cao L Ou D Shen KW Cai G Cai R Xu F. Outcome of Postmastectomy Radiotherapy After Primary Systemic Treatment in Patients With Clinical T1-2n1 Breast Cancer. Cancer Radiotherapie (2018) 22(1):38–44. doi: 10.1016/j.canrad.2017.07.051 29306555

[19] Wang X Xu L Yin Z Wang D Wang Q Xu K. Locoregional Recurrence-Associated Factors and Risk-Adapted Postmastectomy Radiotherapy for Breast Cancer Staged in Ct1-2n0-1 After Neoadjuvant Chemotherapy. Cancer Manag Res (2018) 10:4105–12. doi: 10.2147/CMAR.S173628 PMC6174313 30323666

[20] Wang Q Zhao J Han X Er P Meng X Shi J. Is There a Role for Post-Mastectomy Radiotherapy for T1-2n1 Breast Cancers With Node-Positive Pathology After Patients Become Node-Negative Pathology Following Neoadjuvant Chemotherapy? Front Oncol (2020) 10:892. doi: 10.3389/fonc.2020.00892 32695661 PMC7338570

[21] Zhang Y Zhang Y Liu Z Qin Z Li Y Zhao J. Impact of Postmastectomy Radiotherapy on Locoregional Control and Disease-Free Survival in Patients With Breast Cancer Treated With Neoadjuvant Chemotherapy. J Oncol (2021) 2021:6632635. doi: 10.1155/2021/6632635 33564308 PMC7850833

[22] Kishan AU McCloskey SA. Postmastectomy Radiation Therapy After Neoadjuvant Chemotherapy: Review and Interpretation of Available Data. Ther Adv Med Oncol (2016) 8(1):85–97. doi: 10.1177/1758834015617459 26753007 PMC4699266

