# Supplementary material for: Postmastectomy Radiotherapy After Neoadjuvant Chemotherapy in cT1-2N+ Breast Cancer Patients: A Single Center Experience and Review of Current Literature
Source: Front Oncol. 2022 May 17;12:881047. doi: 10.3389/fonc.2022.881047 (PMC9152099; doi:10.3389/fonc.2022.881047)
Supplement: Supplementary file 1 [file Table_1.docx]

| **Supplementary table 1** Clinical characteristics of patients in the cT_1-2_N_+_ subgroups who achieve ypT_0-2_N_0_ but without PMRT and cT_1-2_N_0_ non-PMRT subgroups. | | | | | | |
| --- | --- | --- | --- | --- | --- | --- |
|  | ypT_0-2_N_0_  non-PMRT | |  | cT_1-2_N_0_  non-PMRT | | *P* value |
| Variable | n=27 | % |  | n=17 | % |  |
| Age |  |  |  |  |  |  |
| Mean | 50.4 |  |  | 47.7 |  |  |
| Range | 33-65 |  |  | 32-62 |  | 1.000 |
| <40 | 3 | 11.1 |  | 2 | 11.8 |  |
| ≥40 | 24 | 88.9 |  | 15 | 88.2 |  |
| Clinical T stage |  |  |  |  |  | 0.121 |
| 1 | 3 | 11.1 |  | 6 | 35.3 |  |
| 2 | 24 | 88.9 |  | 11 | 64.7 |  |
| ypT stage |  |  |  |  |  | 0.064 |
| 0-is | 4 | 14.8 |  | 0 | 0.0 |  |
| 1 | 14 | 51.9 |  | 6 | 35.3 |  |
| 2 | 9 | 33.3 |  | 9 | 52.9 |  |
| Unknown | 0 | 0.0 |  | 2 | 11.8 |  |
| ypN stage |  |  |  |  |  | - |
| 0 | 27 | 100.0 |  | 17 | 100.0 |  |
| 1 | 0 | 0.0 |  | 0 | 0.0 |  |
| 2-3 | 0 | 0.0 |  | 0 | 0.0 |  |
| Unknown | 0 | 0.0 |  | 0 | 0.0 |  |
| Estrogen receptor status |  |  |  |  |  | 0.351 |
| Positive | 10 | 37.0 |  | 9 | 52.9 |  |
| Negative | 14 | 51.9 |  | 8 | 47.1 |  |
| Unknown | 3 | 11.1 |  | 0 | 0.0 |  |
| HER2 status |  |  |  |  |  | 0.079 |
| Positive | 11 | 40.7 |  | 5 | 29.4 |  |
| Negative | 14 | 51.9 |  | 6 | 35.3 |  |
| Unknown | 2 | 7.4 |  | 6 | 35.3 |  |
| TNBC |  |  |  |  |  | 0.574 |
| Yes | 7 | 25.9 |  | 2 | 11.8 |  |
| No | 17 | 63.0 |  | 13 | 76.5 |  |
| Unknown | 3 | 11.1 |  | 2 | 11.8 |  |
| Molecular subtype |  |  |  |  |  | 0.378 |
| Luminal A | 4 | 14.8 |  | 1 | 5.9 |  |
| Luminal B | 5 | 18.5 |  | 8 | 47.1 |  |
| HER2 overexpression | 7 | 25.9 |  | 4 | 23.5 |  |
| Triple-negative | 7 | 25.9 |  | 2 | 11.8 |  |
| Unknown | 4 | 14.8 |  | 2 | 11.8 |  |
| pCR |  |  |  |  |  | 0.056 |
| Yes | 4 | 14.8 |  | 0 | 0.0 |  |
| No | 23 | 85.2 |  | 15 | 88.2 |  |
| Unknown | 0 | 0.0 |  | 2 | 11.8 |  |
| Preoperative chemotherapy regimens |  |  |  |  |  | 0.849 |
| Anthracycline containing | 27 | 100.0 |  | 14 | 82.4 |  |
| Taxane containing | 16 | 59.3 |  | 10 | 58.8 |  |
| Anthracycline and taxane containing | 16 | 59.3 |  | 7 | 41.2 |  |
| Hormone therapy/Estrogen receptor status | |  |  |  |  | - |
|  | 10/10 | 100.0 |  | 9/9 | 100.0 |  |
| HER2-targeted therapy/HER2 status |  |  |  |  |  | 0.728 |
|  | 2/11 | 18.2 |  | 1/6 | 16.7 |  |
| ypT, pathologic tumor size after neoadjuvant therapy; ypN, pathologic lymph node after neoadjuvant therapy; cT, clinical tumor size; cN, clinical lymph node; PMRT, postmastectomy radiotherapy; HER2, human epidermal receptor 2; TNBC, triple negative breast cancer; pCR, pathological complete response. | | | | | | |
