# Supplementary material for: Postmastectomy Radiotherapy After Neoadjuvant Chemotherapy in cT1-2N+ Breast Cancer Patients: A Single Center Experience and Review of Current Literature
Source: Front Oncol. 2022 May 17;12:881047. doi: 10.3389/fonc.2022.881047 (PMC9152099; doi:10.3389/fonc.2022.881047)
Supplement: Supplementary file 2 [file Table_2.docx]

| **Supplementary table 2.** Percentage of patients completing neoadjuvant or total chemotherapy regimen between ypT_0-2_N_0_ PMRT and non-PMRT subgroup | | | |
| --- | --- | --- | --- |
|  | ypT_0-2_N_0_M_0_ | |  |
|  | PMRT | non-PMRT | *P* value |
|  | (n=50) | (n=27) |  |
| Neoadjuvant chemo completion* | 32 (64%) | 9 (33.3%) | **0.010** |
| Total (Neoadjuvant + Adjuvant) chemo completion+ | 48 (96%) | 25(92.6%) | 0.609 |
| *Represents the patient who had completed treatment with neoadjuvant chemotherapy. +Represents the patient who had completed treatment with neoadjuvant and adjuvant chemotherapy. ypT, pathologic tumor size after neoadjuvant therapy; ypN, pathologic lymph node after neoadjuvant therapy; PMRT, postmastectomy radiotherapy. P value in bold indicates statistically significant. | | | |
